# Assessment on induced genetic variability and divergence in the mutagenized lentil populations of microsperma and macrosperma cultivars developed using physical and chemical mutagenesis

**DOI:** 10.1371/journal.pone.0184598

**Published:** 2017-09-18

**Authors:** Rafiul Amin Laskar, Samiullah Khan

**Affiliations:** Mutation Breeding Laboratory, Department of Botany, Aligarh Muslim University, Aligarh, India; National Bureau of Plant Genetic Resources, Pusa India, INDIA

## Abstract

Induced mutagenesis was employed to create genetic variation in the lentil cultivars for yield improvement. The assessments were made on genetic variability, character association, and genetic divergence among the twelve mutagenized populations and one parent population of each of the two lentil cultivars, developed by single and combination treatments with gamma rays and hydrazine hydrates. Analysis of variance revealed significant inter-population differences for the observed quantitative phenotypic traits. The sample mean of six treatment populations in each of the cultivar exhibited highly superior quantitative phenotypic traits compared to their parent cultivars. The higher values of heritability and genetic advance with a high genotypic coefficient of variation for most of the yield attributing traits confirmed the possibilities of lentil yield improvement through phenotypic selection. The number of pods and seeds per plant appeared to be priority traits in selection for higher yield due to their strong direct association with yield. The cluster analysis divided the total populations into three divergent groups in each lentil cultivar with parent genotypes in an independent group showing the high efficacy of the mutagens. Considering the highest contribution of yield trait to the genetic divergence among the clustered population, it was confirmed that the mutagenic treatments created a wide heritable variation for the trait in the mutant populations. The selection of high yielding mutants from the mutant populations of DPL 62 (100 Gy) and Pant L 406 (100Gy + 0.1% HZ) in the subsequent generation is expected to give elite lentil cultivars. Also, hybridization between members of the divergent group would produce diverse segregants for crop improvement. Apart from this, the induced mutations at loci controlling economically important traits in the selected high yielding mutants have successfully contributed in diversifying the accessible lentil genetic base and will definitely be of immense value to the future lentil breeding programmes in India.

## Introduction

The lentil (*Lens culinaris* Medikus subsp. *culinaris*) is a self-pollinated, annual and diploid (2n = 2x = 14) grain legume crop with a relatively large genome of 41063 Mbp [1]. Barulina [2] divided the cultigens *Lens culinaris* Medik. into two sub-species i.e. macrosperma (seed diameter, 6–9 mm) and microsperma (seed diameter, 2–6 mm), later renewed by Cubero [3] as race *macrosperma* and race *microsperma*. Lentil is an excellent dietary staple because of their high protein content and nutrient density that complements the nutritional deficiencies of cereal based diet. Lentil cultivation enhances the soil nutrient status by adding nitrogen, carbon and organic matter. Also, the high market returns of lentil guarantee the economic improvement of rural livelihood. It has also a high level of dietary fiber, vitamin B1, and the straw is valuable animal feed [4].

According to FAO Statistics Division 2014, Asian countries are the biggest lentil producers contributing up to 61% to the total world lentil production. Owing to its large population, most of the biggest consumer and importer countries are also from Asia. The highest production statistics reported in the Asian continent is mainly due to the huge area under lentil cultivation, while continuing low yield potential of the lentil genotypes, especially in South Asia, remain the primary constraint restricting the achievable goal of attaining self-sufficiency. In South Asia, mean yield of Bangladesh, Nepal, India, and Pakistan averaging 914, 857, 652 and 567 kg/ha, respectively is significantly below the world average of 936 kg/ha [5].Since the agricultural land is shrinking day by day in these regions due to high population growth, there is an urgent need to break the key yield constraints, specifically low-yielding cultivated lentil varieties and the narrow genetic base [6]. Erskine *et al*. [7] reported that the lentil germplasm from India exhibits least genetic variability among lentil-producing countries. The adaptation to various stresses through natural selection for high productivity, seed quality, pest and disease resistance and inefficiency of conventional breeding methods exhausted the genetic variability of grain legumes[8]over the course of time, which resulted in limited accessible genetic variability and hence supplemented breeding strategies needs to be incorporated to serve the objective of crop improvement[9].Induced mutations technology, over the past decades, appeared to be one of the most coherent approaches for broadening the genetic base in lentil to circumvent the bottleneck conditions. Also, it is highly recommended especially in lentil due to its tiny flower size which makes recombination breeding a very tedious job. Induced mutagenesis creates anew allelic combination in the desirable traits within a short period of time without disturbing the basic genomic structure of the plant; thereby a sustainable acceleration in creating variation towards desirable direction is possible with very limited risk and resource.

FAO/IAEA Mutant Variety Database records showed that out of total 431 released mutant varieties of legumes, only 13 mutant lentil varieties (two from India) have been released till date. It clearly indicates that an economically important crop like lentil is still not much exploited for mutation breeding. Therefore, work on lentil needs to be accelerated in this direction for developing reproducible induced mutation protocols to create novel genes or new null alleles of genes controlling economically important traits for breeding, especially in India where scope for agricultural intensification is very high. Considering the observations, both microsperma (cv. Pant L 406) and macrosperma (cv. DPL 62) cultivars were subjected to mutagenic treatments of physical (gamma rays) and chemical (hydrazine hydrates) mutagen alone and in combination in the present study, for assessing every possibilities of generating desirable micromutations in the quantitative phenotypic traits of lentil. The yield and yield attributes in lentil are quantitatively inherited polygenic traits [10], and therefore exhibits continuous variations due to the involvement of several independent genes with cumulative effect on the expressivity. Mutations in these traits or micromutations are of small effect that can only be detected with the help of statistical methods. Therefore, different statistical tools were applied to identify the extent of micromutations induced in the different agro-economic traits for selection of best high yielding mutants with other desirable characters. The inheritance pattern and stability of the mutated traits were also accessed in the selected M_2_ and M_3_ mutagenized population. Overall the objectives of present study were to estimate the genetic variation induced by different mutagenic treatments in the quantitative phenotypic traits and their inheritance pattern in the subsequent generation, to assess the genotypic response of microsperma and macrosperma cultivars towards physical, chemical and combination mutagenic treatments, to evaluate the correlation among the different agro-morphological traits with total yield, to compute the heritability and expected genetic gain among the quantitative phenotypic traits achieved in the treated population, to determine the genetic divergence created among the treatment population, to determine the trait contributed maximum to the variation and to identify the potential mutant genotypes for lentil yield improvement through breeding programmes.

## Material and methods

### Plant material

The genetic variability was induced in lentil microsperma cultivar Pant L 406 and macrosperma cultivar DPL 62 (S1 Table) using different treatments of physical mutagen (gamma rays) and chemical mutagen (hydrazine hydrates). The accessions were procured from NBPGR, New Delhi and are highly recommended for the agroclimatic zone of central India.

### Methodology

The healthy and viable seeds (moisture 11.0%) of both the cultivars were directly irradiated with 100(G1), 200(G2), 300(G3) and 400(G4) Gy of gamma rays with a radioisotope 60Co, Cobalt-60, source at the National Botanical Research Institute, Lucknow, Uttar Pradesh, India. The percentage of moisture content was determined following International Seed Testing Association guideline [11], which is based on the difference between fresh weight and dry weight of the seeds. For chemical treatments, presoaked (6 hrs) seeds were treated with different doses (v/v) of hydrazine hydrates (HZ) viz, 0.1%(H1), 0.2%(H2), 0.3%(H3) and 0.4%(H4) at room temperature of 25±2°C for 9 hrs. Also, a combination treatment sets of seeds viz. 100Gyγrays+0.1%HZ (H1+G1), 200Gyγrays+0.2%HZ (H2+G2), 300Gyγrays+0.3%HZ (H3+G3) and 400Gyγrays+0.4%HZ(H4+G4), were prepared by directly treating the gamma treated seeds with hydrazine hydrates concentrations (S2 Table). Initially, the doses of chemical and physical treatments were determined based on LD_50_ values from the germination and survival test. The 300 seeds from each treatment were grown in the agricultural field of Aligarh Muslim University, Aligarh, India during mid-October 2013-April 2014 along with respective controls following a randomized complete block design (RCBD).The total 300 seeds from each treatment were sown in 10 replications of 30 seeds each (3 rows of 10 seeds each per replication). Each block comprised of one replication from each of the 13 treatments i.e., 13 plot (1.8x3 m) per block (1.8x40 m). The spacing was maintained at 0.30 m (seed to seed in a row) and 0.60 m (between the rows)inside each block in the overall field size of 23.5X40meter (S1 Fig).All the self-pollinated fertile M_1_ plants were harvested individually (S2 Table) and 10 healthy M_2_ seeds from each harvested plant were sown in plant progeny row basis for growing M_2_ generation. The quantitative phenotypic traits (S3 Table) data for the statistical study were collected from top 30 plant progeny row means of each treatment that showed excellent survival as well as trait performance. Based on the quantitative statistics for yield,10 M_3_ seeds of selected 30 high yielding plants from each M_2_population viz. H1, H2, G1, G2, H1+G1 and H2+G2populations for cultivar DPL 62 and H2, H3, G2, G3, H1+G1 and H2+G2 populations for cultivar Pant L 406, were considered for propagation into the M_3_ generation. The treatment mean values of ten quantitative phenotypic traits were utilized for statistical analysis to assess the genetic diversity among the mutant population and degree of divergence created by the mutagenic treatments from their respective parents.

### Statistical analysis of quantitative traits

The data on ten quantitative phenotypic traits, namely days to flowering, plant height (cm), days to maturity, number of fertile branches, number of root nodules per plant, number of pods per plant, number of seeds per pod, 100 seed weight per plant (g), total yield per plant (g) and harvest index per plant were recorded throughout M_2_ and M_3_ generations.

Descriptive statistics (mean) associated with different parameters considered in the present investigation across all the concentrations of mutagens used were calculated in M_2_ generation. In order to test the hypothesis that the doses of mutagens used had an effect on the observations made, a between-treatment One-Way ANOVA was performed. Furthermore, to evaluate the nature of the differences between the control and different treatment means, statistically significant ANOVA was followed-up with Duncan Multiple Range Test (DMRT). Character association studies were done by Pearson’s correlation coefficient (*r*) for trait linkage assessment. International Business Machines Corporation Statistical Package for the Social Sciences **(**IBM SPSS 20.0) was used for statistical analysis of the quantitative data.

The genetic parameters were calculated for the traits to predict the extent of induced genetic variability using the formula given by [12], [13]. These parameters include the following:

1. GCV (genotypic coefficient of variation) = $\frac{\sqrt{\sigma 2G}}{x}$ X 100, where,*σ*^2^_G_ (an estimate of genotypic variance) = (MSG −MSE)/*r*; (MSG is an estimate of mean square of tested accession; MSE is an estimate of mean square of error and *r* refers to the number of replications).
2. h^2^bs (an estimate of broad sense heritability) = *σ*^2^_G_/*σ*^2^_P_, where *σ*^2^_G_ is the genotypic component of variance and *σ*^2^_P_ is the phenotypic component of variance.
3. GA (as % of the mean) = (K.σ_P_.h^2^bs/x) X 100, where, h^2^bs = Broad-sense heritability; σ_P_ = Phenotypic standard deviation of the mean performance of treated population; K = 2.64, constant for 1% selection intensity.

In the present experiment, the genetic distance was calculated between the populations of control and treatment M_2_ populations based on the ten phenotypic markers using multivariate methods, to establish the hypothesis that the mutagenic treatments created genetically diverse populations within the two lentil cultivars. The hierarchicalSquared-Euclidean distance matrix-based Cluster analysis was employed based on ten quantitative phenotypic traits of the lentil crop to determine the extent of heterogeneity induced among different treated populations and their respective parents. The Ward’s minimum variance method [14] was used for agglomerative clustering and results were presented graphically in the dendrograms. The scale difference of different quantitative characters was eliminated by recommended range standardization [15], to get the final output with an equal contribution of all the traits. Elbow method based on the agglomerative coefficients was used to determine the number of clusters. The Principal component analysis of the ten quantitative phenotypic traits was performed, to estimate the percentage contribution of each trait to total genetic variation, on a correlation matrix with standardization of original data set due to differences in the trait’s scale and the variation percentage accounted by each principal component was calculated as the eigenvalue divided by the summation of the eigenvalues. These analyses were done using IBM SPSS software version 20.0.

## Results

To assess the extent of induced genetic variability and divergence created by the employed mutagens, the statistical analysis was done on ten quantitative phenotypic traits, namely, days to flowering, plant height, days to maturity, number of fertile branches, number of root nodules per plant, number of pods per plant, number of seeds per pod, 100 seed weight per plant, total yield per plant and harvest index per plant in M_2_ and M_3_ generations of the two cultivars of lentil. The results on the traits and populations from various applied statistical tools are presented below:

### Quantitative traits

In M_2_and M_3_generation, genetic variability in yield with other correlated traits of lentil cultivars DPL 62 and Pant L 406, induced by gamma rays, HZ, and their combination treatments were presented in this section. From the descriptive statistics of different traits in both the cultivars, it can be seen that the lower and medium mutagenic doses were associated with increased mean level of the quantitative phenotypic traits, while the inhibition of expression of traits was maximum in higher treatments compared to their respective control treatments. The independent between treatment groups ANOVA yielded a statistically significant effect with the p value less than 0.05 (p<0.05) in all the quantitative phenotypic traits including grain yield per plant, except days to flowering and seeds per pod, for which the treatments effect was comparatively lower (S4 Table). Thus, the null hypothesis of no differences between the means of different treatment groups rejected and suggested that the mutagen concentrations were successful in creating a range of genetic variations through random mutations. Duncan Multiple Range Test (DMRT) provides the information about the treatment groups deviating significantly from the respective controls. Results of Duncan Multiple Range Test (DMRT) yielded a significant shift in mean values of quantitative phenotypic traits induced by mutagen doses. The trait wise results of the six traits showing maximum genetic variability and high heritability (Tables 1–3) are elaborated below:

**Table 1:** Estimates of mean values, genotypic coefficient of variation (GCV %), broad sense heritability (*h*^*2*^*bs*%) and genetic advance as % of the mean (GA %) for six quantitative traits in the M_2_generation of lentil cultivar DPL 62.

TRAITS		TREATMENT POPULATIONS												
		C	H1	H2	H 3	H4	G1	G2	G3	G4	H1+G1	H2+G2	H3+G3	H4+G4
**Plant Height(cm)**	MEAN^#^	39.93^a^	38.00^c^	37.68^c^	38.73^b^	36.26^e^	38.63^b^	36.49^de^	39.02^b^	35.03^f^	37.52^c^	36.97^d^	36.55^de^	35.19^f^
	GCV %	0.79	2.63	1.98	1.75	1.64	2.27	2.29	1.15	1.04	2.76	1.85	1.61	0.97
	h2%	26.51	51.14	68.31	32.6	27.17	56.73	59.09	35.14	26.35	63.34	54.52	47.4	29.94
	GA%	1.07	4.97	4.32	2.65	2.26	4.52	4.65	1.8	1.41	5.8	3.61	2.92	1.4
**Fertile Branches**	MEAN^#^	8.73^efg^	9.67^bc^	9.97^ab^	9.07^de^	8.47^fg^	10.27^a^	10.47^a^	9.33^cd^	8.67^efg^	10.17^ab^	10.23^ab^	8.97^def^	8.33^g^
	GCV %	5.83	7.86	9.27	6.64	6.38	10.72	8.16	7.04	5.83	10.46	7.43	7.25	5.6
	h2%	28.67	46.7	48.88	34	26.36	59.79	44.11	40.94	27.52	58.26	43.46	37.41	37.95
	GA%	8.24	14.18	17.11	10.22	8.65	21.88	14.31	11.89	8.08	21.08	12.94	11.7	9.11
**Pods per Plant**	MEAN^#^	83.67^e^	86.77^cd^	88.00^ab^	85.90^d^	83.57^e^	88.03^ab^	87.97^ab^	85.73^d^	82.00^f^	87.07^bc^	88.67^a^	88.90^a^	82.63^f^
	GCV %	1.01	2.76	2.32	1.43	2.01	2.41	1.91	1.2	1.36	1.93	1.96	1.91	1.68
	h2%	32.96	67.24	63.09	41.76	53.05	54.79	68.17	34.42	39.74	68.46	52.86	37.95	49.81
	GA%	1.53	5.97	4.86	2.43	3.87	4.7	4.17	1.86	2.27	4.21	3.76	3.11	3.13
**100 Seed Weight (g)**	MEAN^#^	3.14^d^	3.33^a^	3.30^a^	3.29^ab^	3.05^e^	3.31^a^	3.29^a^	3.23^bc^	3.16^cd^	3.22^c^	3.18^cd^	3.13^d^	2.99^e^
	GCV %	1.59	3.57	3.03	1.99	2.15	3.18	2.95	2.84	3.3	3.82	3.13	3.71	2.32
	h2%	17.24	70.15	66.67	46.24	41.75	68.94	65.28	48.28	57.67	68.33	66.44	42.86	44.44
	GA%	1.75	7.88	6.53	3.58	3.67	6.98	6.29	5.2	6.62	8.33	6.73	6.42	4.08
**Grain Yield per Plant (g)**	MEAN^#^	3.85^g^	5.02^a^	4.94^b^	4.61^e^	3.73^h^	4.86^c^	4.74^d^	4.62^e^	3.89^g^	4.68^d^	4.60^e^	4.17^f^	3.55^i^
	GCV %	1.72	2.98	1.97	1.56	1.84	2.15	2.19	1.53	1.8	2.09	2.23	1.9	1.76
	h2%	26.83	65.12	65.52	46.43	37.01	68.55	72.97	45.45	49.49	57.83	63.64	38.65	39.39
	GA%	2.36	6.35	4.22	2.81	2.95	4.7	4.94	2.72	3.34	4.2	4.69	3.12	2.91
**Harvest Index per Plant (%)**	MEAN^#^	28.78^l^	38.28^a^	37.67^b^	35.02^fg^	30.27^j^	36.11^d^	36.70^c^	34.69^g^	32.39^i^	35.70^de^	35.46^ef^	33.32^h^	29.62^k^
	GCV %	1.3	2.53	2.46	2	1.76	4.24	2.06	1.75	2.97	2.39	3.36	2.33	1.96
	h2%	30.34	53.42	52.99	40.31	39.68	72.68	64.83	40.79	42.78	61.53	73.8	48.43	33.27
	GA%	1.9	4.88	4.72	3.35	2.92	9.54	4.39	2.95	5.13	4.95	7.61	4.29	2.98

^#^Means within rows followed by the same letter is not different at the 5% level of significance, based on the Duncan Multiple range test. C = Control; H1 = 0.1%HZ; H2 = 0.2%HZ; H3 = 0.3%HZ; H4 = 0.4%HZ; G1 = 100Gyγrays; G2 = 200Gyγrays; G3 = 300Gyγrays; G4 = 400Gyγrays; H1+G1 = 100Gyγrays+0.1%HZ; H2+G2 = 200Gyγrays+0.2%HZ; H3+G3 = 300Gyγrays+0.3%HZ; H4+G4 = 400Gyγrays+0.4%HZ.

**Table 2:** Estimates of mean values, genotypic coefficient of variation (GCV %), broad sense heritability (*h*^*2*^*bs* %) and genetic advance as % of the mean (GA %) for six quantitative traits in the M_2_ generation of lentil cultivar Pant L 406.

TRAITS		TREATMENT POPULATIONS												
		C	H1	H2	H 3	H4	G1	G2	G3	G4	H1+G1	H2+G2	H3+G3	H4+G4
**Plant Height (cm)**	MEAN^#^	42.69^a^	41.40^b^	40.37^c^	40.18^c^	39.12^d^	41.66^b^	42.99^a^	39.14^d^	38.06^e^	40.42^c^	39.58^d^	39.36^d^	37.95^e^
	GCV %	0.71	1.77	2.48	1.73	1.67	1.2	1.91	2.27	1.1	2.69	1.73	1.36	1.07
	h2%	25.14	36.08	51.15	65.2	30.85	40.44	50.89	61.92	32.37	65.55	54.52	42.75	37.89
	GA%	0.94	2.81	4.68	3.69	2.44	2.02	3.61	4.71	1.65	5.74	3.37	2.34	1.74
**Fertile Branches**	MEAN^#^	7.87^d^	8.33^bcd^	9.23^ab^	8.87^abcd^	8.47^abcd^	8.07^cd^	8.47^abcd^	8.67^abcd^	8.33^bcd^	9.17^abc^	9.47^a^	8.33^bcd^	9.07^abc^
	GCV %	12.73	12.94	16.67	15.73	13.13	12.68	22.93	20.09	12.23	19.08	14.6	10.42	12.25
	h2%	27.84	35.4	53.34	43.45	36.38	37.37	60.97	57.88	35.82	62.63	50.12	33.36	32.24
	GA%	17.73	20.32	32.13	27.38	20.91	20.47	47.26	40.34	19.32	39.86	27.3	15.88	18.36
**Pods per Plant**	MEAN^#^	93.47^de^	95.70^bc^	97.40^a^	96.57^ab^	92.70^e^	95.37^bc^	96.20^abc^	96.50^ab^	92.57^e^	96.57^ab^	96.43^ab^	94.80^cd^	93.67^de^
	GCV %	1.17	1.66	2.55	2.03	1.84	1.55	2.95	2.31	1.66	2.45	2.59	1.82	1.44
	h2%	27.16	39.72	54.07	47.87	35.25	43.77	63.81	64.98	31.13	57.75	61.92	32.03	30.18
	GA%	1.61	2.77	4.95	3.7	2.88	2.71	6.21	4.92	2.45	4.92	5.37	2.72	2.09
**100 Seed Weight (g)**	MEAN^#^	1.92^c^	1.97^abc^	2.00^a^	1.98^abc^	1.93^bc^	1.77^e^	1.97^abc^	1.98^abc^	1.85^d^	1.99^ab^	1.96^abc^	1.76^e^	1.71^e^
	GCV %	2.9	2.87	5.43	4.52	2.88	4.59	5.35	5.42	5.1	5.66	4.56	6.01	3.46
	h2%	21.99	39.02	66.29	61.54	34.07	42.31	68.94	69.7	52.66	64.47	61.54	38.36	36.84
	GA%	3.59	4.74	11.67	9.36	4.45	7.88	11.72	11.94	9.77	12	9.45	9.83	5.54
**Grain Yield per Plant (g)**	MEAN^#^	2.95^f^	3.47^cd^	3.71^a^	3.51^c^	3.06^e^	2.97^f^	3.47^cd^	3.58^b^	3.07^e^	3.41^d^	3.42^d^	2.94^f^	2.56^g^
	GCV %	1.86	1.78	3.76	2.5	1.88	2.38	2.73	2.62	1.95	2.57	2.7	2.33	1.99
	h2%	20	38.78	61.9	60.63	29.2	45.45	64.29	68.75	41.86	52.38	58.62	31.97	30.23
	GA%	2.19	2.92	7.82	5.14	2.68	4.24	5.79	5.74	3.34	4.92	5.45	3.48	2.89
**Harvest Index per Plant (%)**	MEAN^#^	29.81^i^	35.56^c^	38.12^b^	36.71^b^	32.82^f^	30.49^h^	34.45^d^	38.23^a^	33.74^e^	35.06^c^	35.56^c^	31.45^g^	27.76^j^
	GCV %	1.07	1.82	2.41	2.38	1.46	1.82	4.15	1.71	2.69	2.21	3.14	2.25	2.05
	h2%	24	36.56	50.87	50.15	34.71	36.56	69.86	57.94	40.04	57.1	71.25	43.66	32.44
	GA%	1.39	2.9	4.54	4.45	2.26	2.91	9.15	3.44	4.5	4.42	7	3.92	3.08

^**#**^Means within rows followed by the same letter is not different at the 5% level of significance, based on the Duncan Multiple range test. C = Control; H1 = 0.1%HZ; H2 = 0.2%HZ; H3 = 0.3%HZ; H4 = 0.4%HZ; G1 = 100Gyγrays; G2 = 200Gyγrays; G3 = 300Gyγrays; G4 = 400Gyγrays; H1+G1 = 100Gyγrays+0.1%HZ; H2+G2 = 200Gyγrays+0.2%HZ; H3+G3 = 300Gyγrays+0.3%HZ; H4+G4 = 400Gyγrays+0.4%HZ.

**Table 3:** Estimates of mean values, genotypic coefficient of variation (GCV %), broad sense heritability (*h*^*2*^*bs* %) and genetic advance as % of the mean (GA %) for six quantitative traits in the M_3_generation of lentil cultivars DPL 62 and Pant L 406.

TRAITS		TREATMENT POPULATIONS													
		cv. DPL 62							cv. Pant L 406						
		C	H1	H2	G1	G2	H1+G1	H2+G2	C	H 2	H3	G2	G3	H1+G1	H2+G2
**Plant Height (cm)**	MEAN^#^	41.43^a^	38.72^b^	38.11^c^	39.19^b^	37.69^cd^	38.04^c^	37.42^d^	43.15^a^	40.17^c^	41.00^b^	43.13^a^	39.33^d^	40.55^bc^	39.11^d^
	GCV %	0.89	2.72	2.09	2.37	2.35	2.86	1.96	0.83	2.62	1.85	2.27	2.64	3.06	2.14
	h2%	33.17	53.75	71.08	59.48	61.92	65.55	58.01	31.87	53.69	70.04	59.44	69.07	71.24	64.09
	GA%	1.35	5.27	4.65	4.83	4.89	6.1	3.95	1.23	5.07	4.09	4.62	5.8	6.81	4.51
**Fertile Branches**	MEAN^#^	9.17^c^	11.07^b^	11.43^b^	12.23^a^	12.13^a^	11.43^b^	12.27^a^	8.17^b^	9.90^a^	9.17^ab^	9.33^a^	9.67^a^	9.10^ab^	9.43^a^
	GCV %	5.98	6.97	7.92	9.22	8.15	9.77	7.08	12.8	18.42	16.45	20	18.01	19.91	15.27
	h2%	32.45	55.31	64.63	54.22	56.39	61.68	56.66	30.5	76.16	57.56	65.54	57.88	68.85	53.69
	GA%	8.99	10.18	13.5	13.19	12.13	15.9	10.6	18.67	42.44	32.95	42.73	36.17	43.6	29.55
**Pods per Plant**	MEAN^#^	83.53^d^	87.67^c^	89.83^ab^	88.83^b^	89.77^ab^	88.80^b^	90.13^a^	93.80^b^	98.57^a^	98.00^a^	97.97^a^	98.80^a^	98.87^a^	98.17^a^
	GCV %	1.09	2.74	2.23	2.41	1.89	1.84	1.82	1.22	2.53	1.99	2.94	2.28	2.43	2.61
	h2%	36.74	70.66	66.64	62.2	74.78	71.41	57.58	29.71	58.13	61	69.06	73.31	69.29	69.04
	GA%	1.74	6.08	4.8	5.01	4.31	4.11	3.65	1.76	5.08	4.09	6.44	5.15	5.34	5.72
**100 Seed Weight (g)**	MEAN^#^	3.20^b^	3.36^a^	3.41^a^	3.40^a^	3.37^a^	3.35^a^	3.37^a^	1.93^c^	2.02^ab^	2.01^ab^	1.99^b^	2.07^a^	2.04^ab^	2.06^a^
	GCV %	1.93	3.83	3.23	3.39	3.18	3.96	3.25	3.48	5.88	4.98	5.8	5.68	6.02	4.83
	h2%	24.05	73.45	70.76	72.68	69.7	71.54	70.59	29.03	70.15	66.67	72.68	73.4	68.33	66.44
	GA%	2.49	8.68	7.16	7.63	7.01	8.84	7.21	4.94	13	10.72	13.04	12.84	13.14	10.39
**Grain Yield per Plant (g)**	MEAN^#^	4.01^d^	5.21^c^	5.32^b^	5.53^a^	5.44^a^	5.17^c^	5.37^b^	3.00^e^	3.85^b^	3.67^d^	3.64^d^	3.95^a^	3.75^c^	3.70^cd^
	GCV %	1.93	3.07	2.02	2.07	2.1	2.38	2.29	2.39	4.41	2.74	3.03	3.15	2.74	2.91
	h2%	33.33	68	69.88	72.38	76.61	68.33	71.56	29.82	70.59	66.89	70.93	79.49	60	65.71
	GA%	2.94	6.67	4.47	4.65	4.86	5.2	5.11	3.44	9.78	5.91	6.75	7.42	5.6	6.22
**Harvest Index per Plant (%)**	MEAN^#^	29.09^c^	38.43^b^	39.41^a^	39.50^a^	40.07^a^18	38.30^b^	39.69^a^	29.36^f^	38.65^b^	36.82^d^	34.95^e^	40.62^a^	37.91^c^	38.26^bc^
	GCV %	3.71	5.93	6.08	6.1	6.79	4.66	5.51	1.46	3.89	3.06	4.23	2.62	2.45	3.05
	h2%	36.45	51.39	60.57	58.93	64.67	62.65	54.77	36.34	73.48	62.63	71.32	78.4	65.61	72.97
	GA%	2.35	5.93	5.98	9.46	5.48	5.76	7.86	2.33	8.8	6.4	9.44	6.11	5.24	6.87

^**#**^Means within rows in each cultivar followed by the same letter is not different at the 5% level of significance, based on the Duncan Multiple Range Test. C = Control;H1 = 0.1%HZ; H2 = 0.2%HZ; H3 = 0.3%HZ; G1 = 100Gyγrays; G2 = 200Gyγrays; G3 = 300Gyγrays; H1+G1 = 100Gyγrays+0.1%HZ; H2+G2 = 200Gyγrays+0.2%HZ.

#### Plant height (cm)

The recorded data for plant heights in M_2_ generation showed a negative deviation from control mean values against every mutagenic treatment in both the cultivars. Plant height reduced significantly in both DPL 62 and Pant L 406 control population. Maximum reduction was observed with the treatment G4 in DPL 62 and with H4+G4 in Pant L 406. Calculations on GCV% resulted in maximum genotypic variation in cultivar DPL 62 and Pant L 406 with H1+G1. Observation on heritability and genetic advance estimates showed a considerable increase over the control in both the cultivars. Heritability estimates were highest in H2 treatment for cultivar DPL 62 and in H1+G1 treatment for cultivar Pant L 406. The maximum genetic advance was recorded with H1+G1in both the cultivar DPL 62 and Pant L 406.

In anM_3_ generation, the significant reduction in mean plant height was observed in all the treatments of gamma rays and HZ employed alone or in combination in both the cultivars. The reduction in mean plant height was highest in combination treatment of H2+G2 in both the cultivars. The genotypic coefficient of variation, heritability, and genetic advance increased in almost all treatments. The highest genotypic coefficient of variation, heritability, and the genetic advance was recorded in the H1+G1 treated cv. Pant L 406 population. The genetic parameters were similarly affected in both the cultivars.

#### Number of fertile branches

In the M_2_ generation, the mean for a number of fertile branches was shifted towards the positive side in most of the mutagen treatments in both the cultivars. The highest increase in mean was observed in the cv. DPL 62 with G2 and in cv. Pant L 406 with H2+G2.The genotypic coefficient of variation increased considerably in most of the treatments. The estimated GCV was the highest in cv. DPL 62 (G1) whereas, in the cv. Pant L 406, the highest GCV was obtained with G2 treatment. The estimated heritability shows considerable variation for days to maturity and was the highest at G1 (cv. DPL 62) and at H1+G1 (cv. Pant L 406) treatment. The highest genetic advance recorded with G1 treatment in the cv. DPL 62 and with G2 in the cv. Pant L 406.

There was a significant increase in mean values of the fertile branches in all the mutagen treatments in both the cultivars in the M_3_ generation. The population of treatments H2+G2 in cv. DPL 62 and H2 in cv. Pant 406, showed the highest gain in mean values of the fertile branches among all. The genotypic coefficient of variation increased in the mutagenized population. The H2 populations in both the cultivars showed maximum heritability for the trait fertile branches.

#### Number of pods per plant

In the M_2_ generation, the mean shifted mostly in a positive direction with negative deviations in extreme treatments. Significant increases in a mean number of pods per plant were observed in lower and moderate doses of mutagens. The mean values were recorded to be increased more in the cv. DPL 62 with highest in H3+G3 than in the cv. Pant L 406 in H2 compared to respective control mean. The GCV was recorded to be higher with all the treatments of mutagens in both the cultivars. The highest GCV was recorded with H1 (cv. DPL 62) and G2 (cv. Pant L 406) treatments. The calculation on heritability and genetic advance showed considerable variation for the number of pods per plant in both the cultivars. The highest estimated heritability and genetic advance were found in H1+G1 and H1 respectively for cv. DPL 62. The cultivar Pant L 406 showed the maximum heritability and genetic advance withG3 rays and G2 treatments respectively.

The mean values for the number of pods per plant increased over the controls in both the cultivars for all mutagenic treatments selected in the M_3_ generation. The mean values for the treated population differed significantly from the control mean. The genetic parameters were increased in all the treatments of gamma rays and HZ employed alone or in combination. The highest GCV and genetic advance at H1 and heritability at G2 were observed in the cv. DPL 62. The maximum values of genetic parameters for cv. Pant 406 were in G3.

#### 100 seed weight (g)

In the M_2_ generation, the significant shifts in mean values compared to control were observed in both the cultivars. The mean 100 seed weight (g) of cv. DPL 62 and Pant L 406 increased from controls to the maximum in H1 and H2, respectively. The highest GCV was recorded in H1+G1 in the cv. DPL 62 while the highest estimated GCV was obtained at H3+G3 in the cv. Pant L 406. The highest estimated heritability at H1 and genetic advance at H1+G1 were observed in cv. DPL 62, while the cv. Pant L 406 gave the highest estimated heritability and genetic advances with G3.

Mean shift for 100 seed weight (g) were observed to be significantly positive in all the treated populations in the M_3_ generation. The most effective treatments for the increase were H2 and G3 compared to controls in cultivars DPL 62 and Pant L 406, respectively. The genetic parameters were higher in the treated population as compared to controls. The highest GCV and GA % was recorded in combination treatment of H1+G1 and heritability in H1 in the cv. DPL 62, while the highest estimated GCV at H1+G1, heritability % at G3 and GA % at H1+G1 was obtained in the cultivar Pant L 406.

#### Grain yield per plant (g)

The selected M_2_ population from mutagen treatments for rising M_3_ generation exhibited an increase in the mean values for grain yield per plant in both cv. DPL 62 and cv. Pant L 406. The mean values in these treated population significantly differed from that in the control. The mean seed yield of the control populations of cv. DPL 62 and cv. Pant L 406 were increased maximum at H1 and H2, respectively in the treated populations. The genetic parameters for grain yield per plant were also increased in the selected treated population as compared to control. The highest GCV was observed at H1, heritability at G2 and genetic advance at H1 in cv. DPL 62, whereas in the cv. Pant L 406, maximum GCV was at H2, heritability at G3 and genetic advance at H2 were observed.

In M_3_ generation, mutagen treated population, selected for M_3_ generation, exhibited an increase in the mean values for grain yield per plant in both cv. DPL 62 and cv. Pant L 406. The mean values in the treated population significantly increased from that in the control. The grain yield per plant per plant increased from control to maximum at G1 in the cv. DPL 62 while at G3 in cv. Pant L 406. The genetic parameters for grain yield per plant increased in the treated population as compared to control. The highest GCV and GA were reported at H1 and heritability at G2 in the cv. DPL 62. In cv. Pant L 406, the highest GCV and GA at H2 and heritability at G3 were reported.

#### Harvest index per plant (%)

In M_2_ generation, the mean shifted in both positive as well as in negative directions in the treated population. In general, the lower and moderate doses of gamma rays and HZ and the lower doses of gamma rays + HZ combined treatments showed a significant increase in mean yield per plant over the controls in both cv. DPL 62 and cv. Pant L 406. The mean values increased more in the cv. DPL 62 than in the cv. Pant L 406. The genotypic coefficient of variation, heritability, and genetic advance increased over the controls with all the treatments in both the cultivars. In the cv. DPL 62, the highest GCV at G1, heritability at H2+G2 and genetic advance at G1 were recorded highest, whereas GCV and heritability at H2+G2, genetic advance at G2 were highest in cv. Pant L 406. The high heritability coupled with high genetic advance was recorded for a number of the treatments with gamma rays and HZ alone or in combination, indicating that significant gains could be expected from the selection.

Data recorded for harvest index per plant in M_3_ generation showed that the mean values increased significantly with each treatment in both cv. DPL 62 and cv. Pant L 406. The genetic parameters increased in the treated population as compared to the controls. The values of GCV and heritability were highest at G2and GA at G1 treated populations of cv. DPL 62, while in cv. Pant L 406, GCV and GA at G2 and heritability at G3 was highest as compared to their respective control means.

The recorded data on days to flowering, days to maturity, nodules per plant and seeds per pod resulted in non-significant deviation in most of the mutagen doses from the respective controls in both the cultivars DPL 62 and Pant L 406. The mean flowering time was reduced in all the treatments while the mean days to maturity were reduced only in few treated populations. The observation on nodules per plant showed positive deviations mostly in lower and moderate doses of mutagens while number decreases at the highest doses of the mutagens used alone or in combination. The seeds per pod in the both the lentil cultivars revealed that most of the single and combination treatments of gamma rays and HZ were not effective in inducing significant deviations in the mean number of seeds per pod.

### Character association

The results of the character association are given in Table 4. The correlation coefficients results obtained in M_2_ generations showed pods per plant (r = 0.775**, 0.788**), seeds per pod (r = 0.978**, 0.890**) and 100 seed weight (r = 0.936**, 0.894**) had strong correlation with grain yield for the cultivars DPL 62 and Pant L 406, respectively. This suggested that the selection based on these traits in the treated populations can be done for screening high yielding mutants.

**Table 4:** Pearson’s correlation coefficients of different quantitative traits with respect to yield in the two lentil cultivars.

QUANTITATIVE TRAITS	cv. DPL 62	cv. Pant L 406
	Grain yield per plant (g)	Grain yield per plant (g)
Days to flowering	-0.28	-0.11
Plant height (cm)	0.45	0.27
Days to maturity	-0.11	0.16
Fertile branches per plant	0.862**	0.38
Nodules per plant	0.753**	0.715**
Pods per plant	0.775**	0.788**
Seeds per pod	0.978**	0.890**
100 seed weight (g)	0.936**	0.894**
Harvest index per plant (%)	0.962**	0.962**

** Correlation is significant at the 0.01 level (two-tailed).

### Cluster analysis

**Fig 1:**
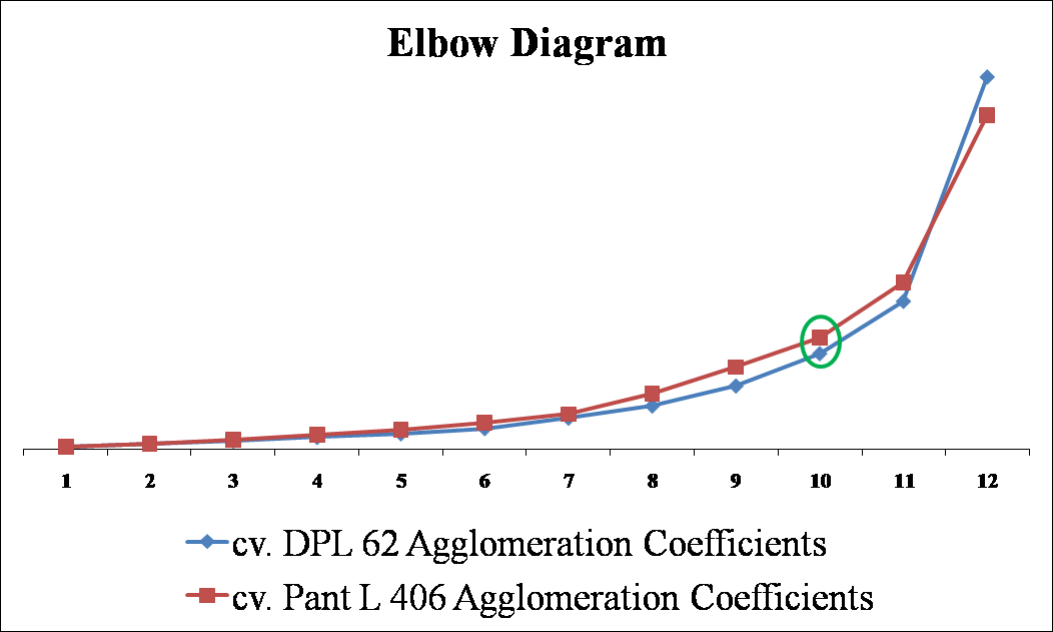
Agglomerative graph of two lentil cultivars for determining number of clusters.

**Fig 2:**
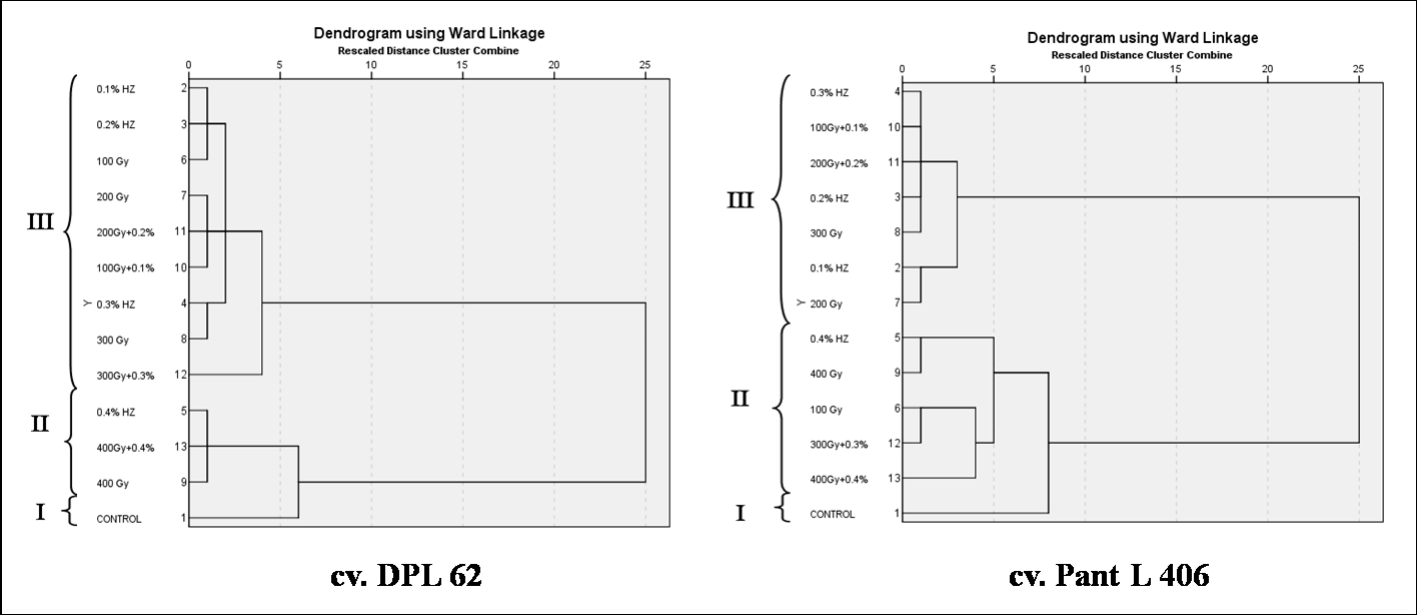
Dendrogram of lentil population clustering based on ten quantitative traits.

The 12 treated populations and 1 control population from each cv. DPL 62 and Pant L 406 were clustered based on the ten quantitative phenotypic traits recorded. The number of clusters was determined to be three in the population of both the cultivars by the agglomerative coefficient graph using elbow method (Fig 1). The phylogenetic relationships among the populations were presented in Dendrogram (Fig 2) for both the cultivars. The clustering pattern of different populations in the dendrogram showed the dissimilarity between the clusters as well as the hierarchical categorization within and among clusters. The treatment population 0.1% HZ in the case of cv. DPL 62 and 0.3% HZ in the case of cv. Pant L 406 were found to be the most mutated population with respect to their respective controls. The observed populations of the two lentil cultivar formed three clusters each with cluster size 1-9in cv. DPL 62 and 1–7 in cv. Pant L 406. It was significant to note that in both the cultivars the control population formed an independent cluster, which showed that considerable genetic variations were created in the quantitative phenotypic traits of the cultivars due to mutagenic treatments. The maximum number of treated populations was included in the Cluster III having 9 and 7 treatment population of the cv. DPL 62 and cv. Pant L 406, respectively. The interpopulation dissimilarity matrix revealed that highest squared Euclidean distance is between the population of H4+G4 and H2 in both the cultivar DPL 62 (5.50) and Pant L 406 (5.26) while lowest is between the population H1 and H2 in DPL 62 (0.15) and H1+G1 and H3 in Pant L 406 (0.17). The most distanced population from control population is the H1 (4.78) in cv. DPL 62 and H2 (4.98) in cv. Pant L 406 (Table 5). The highest inter-cluster distance was observed between cluster I and III in both the cultivar DPL 62 (10.287) and Pant L 406 (7.459) (Table 6). The cluster mean of the three distant clusters for the ten quantitative phenotypic traits are presented in Table 7. The Cluster III had the highest mean values for yield per plant (4.69 and 3.51) and harvest index per plant (35.88% and 36.24%) in cv. DPL 62 and cv. Pant L 406, respectively. Hence, the population falling under this cluster could be screened for the selection of high yielding plants.

**Table 5:** Interpopulation proximity matrix based on squared Euclidean distance in the different populations of cv. DPL 62 and cv. Pant L 406.

		C	H1	H2	H3	H4	G1	G2	G3	G4	H1+G1	H2+G2	H3+G3	H4+G4	
C	**DPL62**		2.33	**4.98**	3.40	1.67	1.73	2.68	4.19	2.86	3.72	4.26	2.80	3.97	**Pant L 406**
H1		**4.78**		0.71	0.36	1.25	0.98	0.22	0.47	1.54	0.65	0.81	1.40	3.73	
H2		4.51	***0*.*15***		0.27	2.84	2.65	0.87	0.34	3.01	0.48	0.42	2.76	**5.26**	
H3		2.65	0.48	0.63		1.99	1.74	0.53	0.25	2.32	***0*.*17***	0.35	1.91	4.14	
H4		1.83	3.82	3.85	2.01		1.24	1.95	1.90	0.26	2.12	1.85	0.98	1.61	
G1		4.34	0.30	0.28	0.62	3.53		1.10	2.00	1.34	1.81	1.95	0.45	1.84	
G2		4.98	0.51	0.27	0.96	3.46	0.33		0.77	2.37	0.63	0.90	1.64	4.14	
G3		2.78	0.52	0.58	0.24	2.11	0.45	0.83		1.88	0.56	0.42	1.70	4.15	
G4		2.45	3.37	3.63	1.87	0.43	3.44	3.28	2.33		2.54	2.02	0.72	1.48	
H1+G1		3.75	0.36	0.22	0.52	2.66	0.21	0.19	0.32	2.66		0.21	1.90	3.60	
H2+G2		4.67	0.82	0.48	1.03	2.99	0.51	0.19	0.71	3.32	0.21		1.60	3.05	
H3+G3		3.70	2.08	1.73	1.40	1.59	1.66	1.28	1.15	2.44	1.16	0.74		0.99	
H4+G4		3.07	5.37	**5.50**	3.35	0.24	4.96	4.75	3.37	0.72	3.95	4.17	2.33		

C = Control; H1 = 0.1%HZ; H2 = 0.2%HZ; H3 = 0.3%HZ; H4 = 0.4%HZ; G1 = 100Gyγrays; G2 = 200Gyγrays; G3 = 300Gyγrays; G4 = 400Gyγrays; H1+G1 = 100Gyγrays+0.1%HZ; H2+G2 = 200Gyγrays+0.2%HZ; H3+G3 = 300Gyγrays+0.3%HZ; H4+G4 = 400Gyγrays+0.4%HZ.

**Table 6:** Intercluster distances among the lentil populations.

		CLUSTER I	CLUSTER II	CLUSTER III		
CLUSTER I	**DPL 62**		4.938	7.459	**Pant L 406**	CLUSTER I
CLUSTER II		6.691		6.987		CLUSTER II
CLUSTER III		10.287	8.799			CLUSTER III

**Table 7:** Characteristic means of three cluster groups of thirteen lentil populations.

QUANTITATIVE TRAITS	DPL 62			Pant L 406		
	CLUSTER I	CLUSTER II	CLUSTER III	CLUSTER I	CLUSTER II	CLUSTER III
Days to flowering	79.00	76.33	76.78	83.23	80.65	80.84
Plant height (cm)	39.93	35.49	37.73	42.69	39.23	40.58
Days to maturity	131.00	127.67	127.56	138.83	135.21	135.79
Fertile branches per plant	9.00	8.33	9.67	7.87	8.45	8.89
Nodules per plant	20.00	19.33	24.11	24.3	24.74	27.77
Pods per plant	84.00	83.00	87.56	93.47	93.82	96.48
Seeds per pod	1.00	1.33	2.00	1.63	1.72	1.83
100 seed weight (g)	3.14	3.07	3.25	1.92	1.80	1.98
Grain yield per plant (g)	3.85	3.72	**4.69**	2.95	2.92	**3.51**
Harvest index per plant(%)	28.78	30.76	35.88	29.81	31.25	36.24

### Principle component analysis (PC)

The traits which contributed in separating the different treated population are presented in Table 8. The group traits with the highest positive and negative loading were shown in bold and italic fonts, respectively. The largest group with positive loading on PC1 is the yield (0.9811 and 0.9653) while the largest group with negative loading on PC1 is days to flowering (-0.3377 and -0.2356), in cultivars DPL 62 and Pant L 406, respectively. For PC2, days to maturity (0.8599 and 0.8930) showed positive loading in cultivars DPL 62 and Pant L 406, respectively. The two extracted principal components PC1and PC2 from the original data have the latent roots (Eigen value) more than one, thereby accounting approximately 84% and 79% of the total variation in the cultivars DPL 62 and Pant L 406, respectively. The first PC, in the present experiment, summarized most of the variability viz. 61.35% (DPL 62) and 51.47% (Pant L 406) present in the original mean data collected from M_2_ treated population relative to remaining PC. The second PC explained the most of the variability viz. 22.87% (DPL 62) and 27.80% (Pant L 406) that was uncorrelated and not summarized by the first PC. Therefore, the total variation in the original data set is broken down into independent components and their cumulative variation % was observed to be 84.24% and 79.27 in cv. DPL 62 and cv. Pant L 406, respectively. The PCA analysis revealed that the yield contributed maximum towards divergence in the populations, therefore, confirmed that the yield trait responded desirably to the mutagenic treatments for possible selection of the high yielding mutants. The loading plot of the biometrical traits in first two PCs clearly showed that the yield related traits had contributed considerably towards the overall genetic variation. The distribution pattern also indicated the existence of the significant amount of variability among the mutagenized population for the observed quantitative traits.

**Table 8:** Principal components analysis showing the contribution of ten quantitative traits among the lentil populations.

TRAITS	PRINCIPAL COMPONENTS (PCS)			
	DLP 62		Pant L 406	
	PC 1	PC 2	PC 1	PC 2
Days to flowering (Days)	*-0*.*3377*	0.8247	*-0*.*2356*	0.8837
Plant height (cm)	0.3638	0.8297	0.2005	0.8244
Days to maturity (Days)	-0.2532	**0.8599**	-0.0148	**0.8930**
Fertile branches per plant	0.9030	-0.0081	0.5483	*-0*.*5800*
Nodules per plant	0.8483	*-0*.*1394*	0.8504	-0.0241
Pods per plant	0.8703	-0.1353	0.8981	-0.0107
Seeds per pod	0.9365	0.1462	0.8826	-0.0628
100 seed weight (g)	0.8776	0.3070	0.8059	0.3916
Grain yield per plant (g)	**0.9811**	0.1289	**0.9653**	0.1657
Harvest index per plant (%)	0.9605	-0.0989	0.9274	-0.0170
EIGENVALUE	6.1354	2.2878	5.1466	2.7801
% OF VARIANCE	61.3541	22.8778	51.4658	27.8012
% CUMULATIVE OF VARIANCE	61.3541	84.2319	51.4658	79.2671

## Discussion

### Genetic variability analysis

The wide genetic variability is prerequisite for initiate a breeding programme. The extent of genetic variation present in the crop genepool determines the success of breeding. Induce mutation technology have been proved to be best among the available techniques for creating a novel genetic blend in crop genome. It facilitates the plant breeders to screen and select the desired combination of expressed economic traits. The study of induced micromutations is an important and tedious work to be accomplished and needs extensive analysis of the yield related quantitative traits by statistical tools for selecting the best improved mutant genotype. It is evident from the several reported works in various crops [16], [17], [18],[19],[20],[21],[22] that considerable genetic variability in the mutagenized population is due to induced micromutations. It was found in the studies of induced mutations for quantitative traits improvement that responses vary from trait to trait towards similar mutagenic treatments, therefore, doses and durations of the mutagenic treatments generates different responses from different traits [23], [24], [25],. Moreover, the genotypic background of the plant material under study governs the direction of micromutations [26].

Therefore, to devise an appropriate breeding strategy and to predict the maximum genetic improvement for a particular trait that can be achieved through selection, the estimations of genetic parameters like genotypic coefficient of variation (GCV %), heritability (h^2^) and genetic advance (GA %) are needed. The observation on different traits revealed a wide extent of inter-traits variability is induced by the different treatments of gamma rays and hydrazine hydrates alone or in combination. Study on the shift in mean values of different quantitative traits showed mostly desirable shifts in different yield contributing traits with few insignificant exceptions where mutagenic treatments reduce the mean values. Since the large numbers of genes are involved in the expression of quantitative traits and considering the randomness with which mutagen interact with the targeted genome, the variations in both directions from the control means are very much expected. In the study, the analysis of variance using genetic parameters indicated significant variation for all six quantitative traits among the different mutagenized population compared to their control population, confirming the possibility for improvement through selection in the subsequent generations. The higher values of heritability resulted from narrow differences between phenotypic and genotypic coefficients of variation for most of the selected treatment populations in M_3_ generation showed minimum environmental variances at the expression level of the polygenic traits in both the lentil cultivars. The values for heritability and genetic advances showed an independent trend for most of the treatment populations in most of the quantitative traits which revealed the fact that high heritability and large genetic advance could not be expected always [27], [28]. Therefore, Ogunniyan and Olakojo[29] recommended that consideration of heritability in association with the genetic advance in plant breeding would facilitate the effective prediction of elite genotype selection outcome. The improvement in the genetic parameters from M_2_ to M_3_ generations in the propagated mutant populations established the efficacy of the present selection lines. It can be inferred from the present study that induced micromutations are random events and extent or direction of induced mutations are completely governs by the type, doses, and durations of mutagens and the targeted genotype/trait. This notion was also supported by other workers[30], [31]. Rizwan *et al*. [32] and many other pieces of literature are available on the induction mutation in lentil cultivars using different mutagenic agents to generate genetically divergent populations within the individual parental populations for selection of desirable mutants based on the breeding objective. The result of the quantitative data analysis showed that macrosperma cultivar DPL 62 responded desirably for most of the agro-morphological traits towards the initial two single treatments, whereas microsperma cultivar Pant L 406 responded similarly towards the medium two single treatments of gamma rays and HZ, while sensitivity towards combination treatments was found to be in comparable trend. The higher combination treatments generated heavy mutations, mostly undesirable, due to the synergistic effect of the both on the crop genetic material. It is recommended to apply combination treatments one after other to avoid the highly competitive nature of the mutagens towards the genetic material and to create a chance for the next mutagen to affect the part of the DNA that was not attacked by the first one, so possibility of more number of mutations is higher in case of combination treatments, which sometimes also resulted in negative impact. The enlargement in the range of variability by the mutagenic treatments for yield and its attributes such as fertile branches, pods per plant and 100 seed weight for the two lentil cultivars in M_2_ and M_3_ generations of the present study has confirmed the ample possibilities for selection of elite mutants in the subsequent generations.

### Character association analysis

The degree of the linear relationship between two traits considered to be the function of selection, gene linkage, and pleiotropy [33]. The characterization of the 13 population in each of the two lentil cultivar was done based on the correlation coefficients of ten quantitative traits. The estimated correlation coefficients of the traits are presented in Table 4. The high positive and significant correlation value with yield was obtained. The strength of trait correlation with yield was found to be seeds per pod>harvest index (%)>100 seed weight (g) in cv. DPL 62 and harvest index (%)>100 seed weight (g)>seeds per pod in cv. Pant L 406. The yield was also positively and significantly associated with number of pods, nodules and fertile branches. The strength of correlation was observed to stronger in cv. DPL 62 compared to cv. Pant L 406. The correlated traits could contribute to the quantity of food synthesized by the plant during photosynthesis or plant physiological activities and thereby have a direct impact on yield. It is evident from the result that the selection in any one of these inter-correlated yield attributing traits will lead to increase in the grain yield. Hence, selection for these traits should also be given considerable importance along with yield during the mutation breeding experiment on lentil [34], [6].

### Multivariate analysis of genetic divergence

Genetic distance is defined as “any quantitative measure of genetic difference, be it at the sequence level or the allele frequency level that is calculated between individuals, populations or species” [35]. Phenotypic characterization of the mutant population with respect to the parents is very important part of mutation breeding programme and effective evaluation of the different interrelated traits, especially quantitative traits is the key to successful selection. Simultaneous evaluation of many phenotypic traits during the crop development leads to uncertainty and error in selection. The application of multivariate statistical algorithms in mutation breeding is of significant importance for classifying the diverse induced mutant populations according to their genetic relationship. The use of statistical grouping tools for the identification and classification of mutants originated from same parents was also suggested [36]. The Multivariate analytical techniques like cluster analysis and principal component analysis do the simultaneous analysis of multiple measurements on each individual population for the genetic diversity among them irrespective of the data set (morphological, chemical, or molecular marker data). It is also noteworthy to mention that each of the data set has its own strengths and constraints and there is no single or simple strategy to effectively analyze the genetic diversity at various levels (individuals, populations, or species). The cluster analysis and principal component analysis as a key statistical tool for sorting out multiple interrelated population in plant breeding was also reported by many authors [37], [38]. In the present work, multivariate analysis was undertaken primarily to estimate the induced diversity created in the mutagenized population compared to the control and the percentage of variation explained by the yield trait to confirm the possibility of selection on the basis of yield trait. Multivariate analysis is basically a data reduction technique to improve breeding accuracy, therefore, the cluster analysis and principal component analysis were used to simplify data for selection. The cluster analysis of the treated population with respective control populations was performed to group the populations based on their responses to the ten phenotypic markers i.e., based on distance or proximity. The principal component analysis was done to form the group of phenotypic markers based on the different population’s responses to them i.e., based on patterns of variation (correlation).

The inter-cluster distance is useful in determining the parental line in hybridization program and the higher distance between the clusters will give a broad spectrum of variability in the segregating generation. Rohman *et al*., [39] suggested that the highest contributing clusters to the divergence should be given greater prominence to choose the cluster type for further selection and parents in subsequent hybridization. In the mutation breeding, the most distinct cluster with respect to the parental cluster can be effectively used for mutant selection in the subsequent generations. The cluster analysis employed in the present breeding experiment divided the mutant populations into different clusters, which were deviated significantly from the respective controls, thereby revealed that the mutagenic treatments induced heterogeneous populations in the two parental lines used. Also, the members within the clusters are genetically closer and members in different clusters are farther apart (Fig 2).

In the present work, Principal component analysis of the yield related traits of M_2_ populations was done to categorize the representative traits for phenotypic characterization of the mutated lentil populations, and thereby to identify superior high yielding plants for propagation into next M_3_ generation. The reduced numbers of the uncorrelated variable (PCs) are resulted by linear transformation of the original variables in the data set. The major contributors to the genetic divergence were found to be yield per plant and harvest index per plant; therefore, further selection on these traits in the subsequent generation may possibly lead to the isolation of more diverse yield trait mutants. Afuape *et al*. [40] also suggested that the PCA can be highly effective in the selection of desirable lines for further breeding purpose. PCA is successful in depicting relative contribution of characters which led to observed genotypic divergence i.e., the high contribution of few characters or small contribution each character.

## Conclusion

The study suggests that quantitative phenotypic markers are the useful tool for preliminary assessment of genetic diversity. The high GCV together with high heritability and genetic advance resulted from the mutagenic treatments in the selected mutagenized populations compared to respective parents showed the wide possibility of selection for improving yield trait in the lentil cultivars. The character association studies clearly showed that the significant and positive correlation of pods per plant, seeds per pod and 100 seed weight (g) with yield. The hierarchical cluster analysis grouped the population of treatments and controls into three primary clusters in the lentil cultivars DPL 62 and pant L 406. Hence, selection of mutants must be based on the wider inter-cluster distance with the controls and superior mean performance for yield and yield components. The results of the assessment on induced micromutations for the yield trait indicated significant inter-population divergence have been created by the different mutagenic treatments. Based on the mean quantitative trait data and genetic parameters, six mutagenized population, namely, 0.2% HZ (H2); 100 Gy γ rays (G1); 200 Gy γ rays (G2); 200 Gy γ rays + 0.2% HZ (H2+G2) in cv. DPL 62 and 0.2% HZ (H2); 300 Gy (G3) in cv. Pant L 406, was found to be superior for grain yield in the M_3_ generation. Therefore, the high yielding mutant selection from these populations should be considered in future breeding program for developing elite mutant cultivars. Also, the divergent populations could be used for hybridization program for obtaining desirable segregants in the subsequent generations.

## Supporting information

S1 Table Description of lentil genotypes used in present study. (DOCX)

S2 Table List of different traits and their description of measurement. (DOCX)

S3 Table Description of M_2_ populations of lentil derived through induced mutagenesis. (DOCX)

S4 Table Significance in terms of probability (p) of *F*-test for the analysis of variance (ANOVA) for each of the ten traits of lentil cultivars evaluated in M_2_ generation. Df = Degree of freedom, ** = at p < 0.05 level significance, ns = not significant, based on the One-way ANOVA analysis.
(DOCX)

S1 Fig Field plots layout of M_1_ generations in a randomized complete block design (RCBD) for each cultivar. (TIF)

S2 Fig (i) Experimental field. (ii) Seeds of lentil microsperma cv. Pant L 406. (iii) Seeds of lentil macrosperma cv. DPL 62. (iv) Fertile “dwarf” mutant (v) Fertile “tall” mutant (vi) Fertile normal plant. (vii) Fertile “semi-dwarf “mutant. (viii) Fertile “high yielded bushy” mutant. (ix) Fertile “high yielded tall” mutant. (x) Multiple pods per peduncle.
(TIF)
