# Supplementary material for: Assessment on induced genetic variability and divergence in the mutagenized lentil populations of microsperma and macrosperma cultivars developed using physical and chemical mutagenesis
Source: PLoS One. 2017 Sep 18;12(9):e0184598. doi: 10.1371/journal.pone.0184598 (PMC5603160; doi:10.1371/journal.pone.0184598)
Supplement: S1 Table — (DOCX) [file pone.0184598.s001.docx]

**S1 Table.** Description of lentil cultivars used in present study.

| **Sl. No.** | **Cultivar** | **Pedigree** | **Place and year of origin** |
| --- | --- | --- | --- |
| 1 | DPL 62 | JLS 1 X LG 171 | GBPUA&T, Pantnagar, 1979 |
| 2 | Pant L 406 | Selection P-495 | IIPR, Kanpur, 1996 |
