# Supplementary material for: Assessment on induced genetic variability and divergence in the mutagenized lentil populations of microsperma and macrosperma cultivars developed using physical and chemical mutagenesis
Source: PLoS One. 2017 Sep 18;12(9):e0184598. doi: 10.1371/journal.pone.0184598 (PMC5603160; doi:10.1371/journal.pone.0184598)
Supplement: S2 Table — (DOCX) [file pone.0184598.s002.docx]

**S2 Table.** Description of M_2_ populations of lentil derived through induced mutagenesis.

| **Population Code** | **Pedigree/**  **Treatments** | **Total no. of fertile plants in M_1_ Generation** | **Total no. of plants survived in M_2_ Generation** |
| --- | --- | --- | --- |
| **cv. DPL 62** | | | |
| C | Parent | 269 | 2495 |
| G1 | 100 Gy | 236 | 2118 |
| G2 | 200 Gy | 215 | 1816 |
| G3 | 300 Gy | 203 | 1620 |
| G4 | 400 Gy | 186 | 1370 |
| H1 | HZ 0.1% | 243 | 2193 |
| H2 | HZ 0.1% | 225 | 1902 |
| H3 | HZ 0.1% | 211 | 1666 |
| H4 | HZ 0.1% | 193 | 1430 |
| H1+G1 | 100Gyγrays+0.1%HZ | 220 | 1856 |
| H2+G2 | 200Gyγrays+0.2%HZ | 198 | 1538 |
| H3+G3 | 300Gyγrays+0.3%HZ | 181 | 1321 |
| H4+G4 | 400Gyγrays+0.4%HZ | 175 | 1204 |
| **Pooled Total** | | **2755** | **22529** |
| **Pant L 406** | | | |
| C | Parent | **260** | **2402** |
| G1 | 100 Gy | 225 | 1916 |
| G2 | 200 Gy | 208 | 1689 |
| G3 | 300 Gy | 192 | 1431 |
| G4 | 400 Gy | 172 | 1186 |
| H1 | HZ 0.1% | 217 | 1860 |
| H2 | HZ 0.1% | 198 | 1524 |
| H3 | HZ 0.1% | 184 | 1297 |
| H4 | HZ 0.1% | 166 | 1081 |
| H1+G1 | 100Gyγrays+0.1%HZ | 201 | 1533 |
| H2+G2 | 200Gyγrays+0.2%HZ | 181 | 1292 |
| H3+G3 | 300Gyγrays+0.3%HZ | 163 | 1107 |
| H4+G4 | 400Gyγrays+0.4%HZ | 155 | 1002 |
| **Pooled Total** | | **2522** | **19320** |

| TREATMENT 01 | | | | | | | | | | | | | | | | | | | | | | | | | | | | | | |
| --- | --- | --- | --- | --- | --- | --- | --- | --- | --- | --- | --- | --- | --- | --- | --- | --- | --- | --- | --- | --- | --- | --- | --- | --- | --- | --- | --- | --- | --- | --- |
| REPLICATIONS | | | | | | | | | | | | | | | | | | | | | | | | | | | | | | |
| R | 1 | | | 2 | | | 3 | | | 4 | | | 5 | | | 6 | | | 7 | | | 8 | | | 9 | | | 10 | | |
|  | 30 | | | 30 | | | 30 | | | 30 | | | 30 | | | 30 | | | 30 | | | 30 | | | 30 | | | 30 | | |
| M1 | 10 | 10 | 10 | 10 | 10 | 10 | 10 | 10 | 10 | 10 | 10 | 10 | 10 | 10 | 10 | 10 | 10 | 10 | 10 | 10 | 10 | 10 | 10 | 10 | 10 | 10 | 10 | 10 | 10 | 10 |
| M2 | Survive | | | Survive | | | Survive | | | Survive | | | Survive | | | Survive | | | Survive | | | Survive | | | Survive | | | Survive | | |
|  | Raws | | | Raws | | | Raws | | | Raws | | | Raws | | | Raws | | | Raws | | | Raws | | | Raws | | | Raws | | |
