# Supplementary material for: Assessment on induced genetic variability and divergence in the mutagenized lentil populations of microsperma and macrosperma cultivars developed using physical and chemical mutagenesis
Source: PLoS One. 2017 Sep 18;12(9):e0184598. doi: 10.1371/journal.pone.0184598 (PMC5603160; doi:10.1371/journal.pone.0184598)
Supplement: S3 Table — (DOCX) [file pone.0184598.s003.docx]

**S3 Table.** List of different phenotypic traits and their quantitative measurement description.

| **Sl. No.** | **Traits** | **Method of measurement** |
| --- | --- | --- |
| 1 | Days to flowering | days taken from sowing to date of opening of first flower |
| 2 | Days to maturity | days taken from sowing until the date of harvesting |
| 3 | Plant height (cm) | height from the base up to the apex of the plant |
| 4 | Branches per plant (number) | number of pod bearing branches at maturity |
| 5 | Nodules per plant (number) | number of root nodules in the uprooted plant |
| 6 | Pods per plant (number) | total number of pods per plant with seed |
| 7 | Seeds per pod (number) | number of seeds in a pod |
| 8 | 100-seed wt (g) | weight of a random sample of 100 seeds |
| 9 | Seed yield per plant (g) | weight of total number of seeds harvested |
| 10 | Harvest Index (%) | the ratio of grain yield to biological yield |
