# Supplementary material for: Assessment on induced genetic variability and divergence in the mutagenized lentil populations of microsperma and macrosperma cultivars developed using physical and chemical mutagenesis
Source: PLoS One. 2017 Sep 18;12(9):e0184598. doi: 10.1371/journal.pone.0184598 (PMC5603160; doi:10.1371/journal.pone.0184598)
Supplement: S4 Table — Df = Degree of freedom, ** = at p < 0.05 level significance, ns = not significant, based on the One-way ANOVA analysis. (DOCX) [file pone.0184598.s004.docx]

**S4 Table.** Significance in terms of probability (p) of *F*-test for the analysis of variance (ANOVA) for each of the ten traits of lentil cultivars evaluated in M_2_ generation.

| **Traits** | **Source of Variation**  **(Treatments)** | **df** | **cv. DPL 62** | | | **cv. Pant L 406** | | |
| --- | --- | --- | --- | --- | --- | --- | --- | --- |
|  |  |  | **Mean Square** | **F** | **p** | **Mean Square** | **F** | **p** |
| Days to flowering | Between | 12 | 19.01 | 0.84 | 0.61^ns^ | 16.46 | 0.73 | 0.72^ns^ |
|  | Within | 377 | 22.71 |  |  | 22.61 |  |  |
| Plant height (cm) | Between | 12 | 65.68 | 76.26 | 0.00^**^ | 76.62 | 87.14 | 0.00^**^ |
|  | Within | 377 | 0.86 |  |  | 0.88 |  |  |
| Days to maturity | Between | 12 | 52.93 | 16.52 | 0.00^**^ | 46.55 | 11.66 | 0.00^**^ |
|  | Within | 377 | 3.20 |  |  | 3.99 |  |  |
| Fertile branches per plant | Between | 12 | 17.14 | 15.44 | 0.00^**^ | 7.04 | 2.10 | 0.02 |
|  | Within | 377 | 1.11 |  |  | 3.36 |  |  |
| Nodules per plant | Between | 12 | 162.36 | 46.58 | 0.00^**^ | 106.32 | 33.54 | 0.00^**^ |
|  | Within | 377 | 3.49 |  |  | 3.17 |  |  |
| Pods per  plant | Between | 12 | 169.92 | 40.03 | 0.00^**^ | 79.39 | 11.39 | 0.00^**^ |
|  | Within | 377 | 4.24 |  |  | 6.97 |  |  |
| Seeds per  pod | Between | 12 | 0.32 | 1.07 | 0.39^ns^ | 0.23 | 0.63 | 0.82^ns^ |
|  | Within | 377 | 0.30 |  |  | 0.36 |  |  |
| 100 seed weight (g) | Between | 12 | 0.34 | 24.52 | 0.00^**^ | 0.30 | 23.57 | 0.00^**^ |
|  | Within | 377 | 0.01 |  |  | 0.01 |  |  |
| Grain yield  per plant (g) | Between | 12 | 7.50 | 587.66 | 0.00^**^ | 3.38 | 290.18 | 0.00^**^ |
|  | Within | 377 | 0.01 |  |  | 0.01 |  |  |
| Harvest index per plant (%) | Between | 12 | 282.90 | 242.16 | 0.00^**^ | 312.06 | 284.88 | 0.00^**^ |
|  | Within | 377 | 1.17 |  |  | 1.10 |  |  |

Df = Degree of freedom, ** = at p < 0.05 level significance, ns= not significant, based on the One-way ANOVA analysis.
