# Supplementary figures and images for: Assessment on induced genetic variability and divergence in the mutagenized lentil populations of microsperma and macrosperma cultivars developed using physical and chemical mutagenesis
Source: PLoS One. 2017 Sep 18;12(9):e0184598. doi: 10.1371/journal.pone.0184598 (PMC5603160; doi:10.1371/journal.pone.0184598)

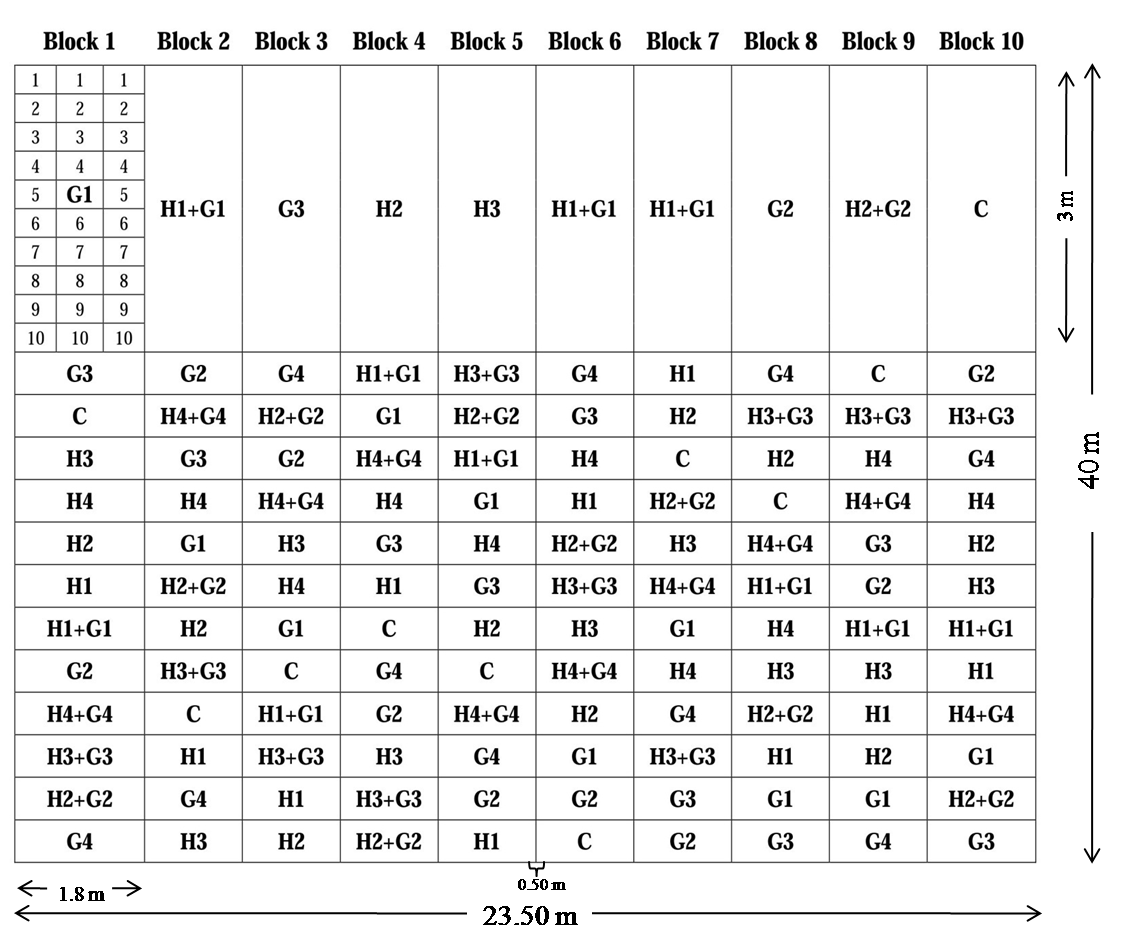

Supplement: S1 Fig — (TIF) [file pone.0184598.s005.tif]

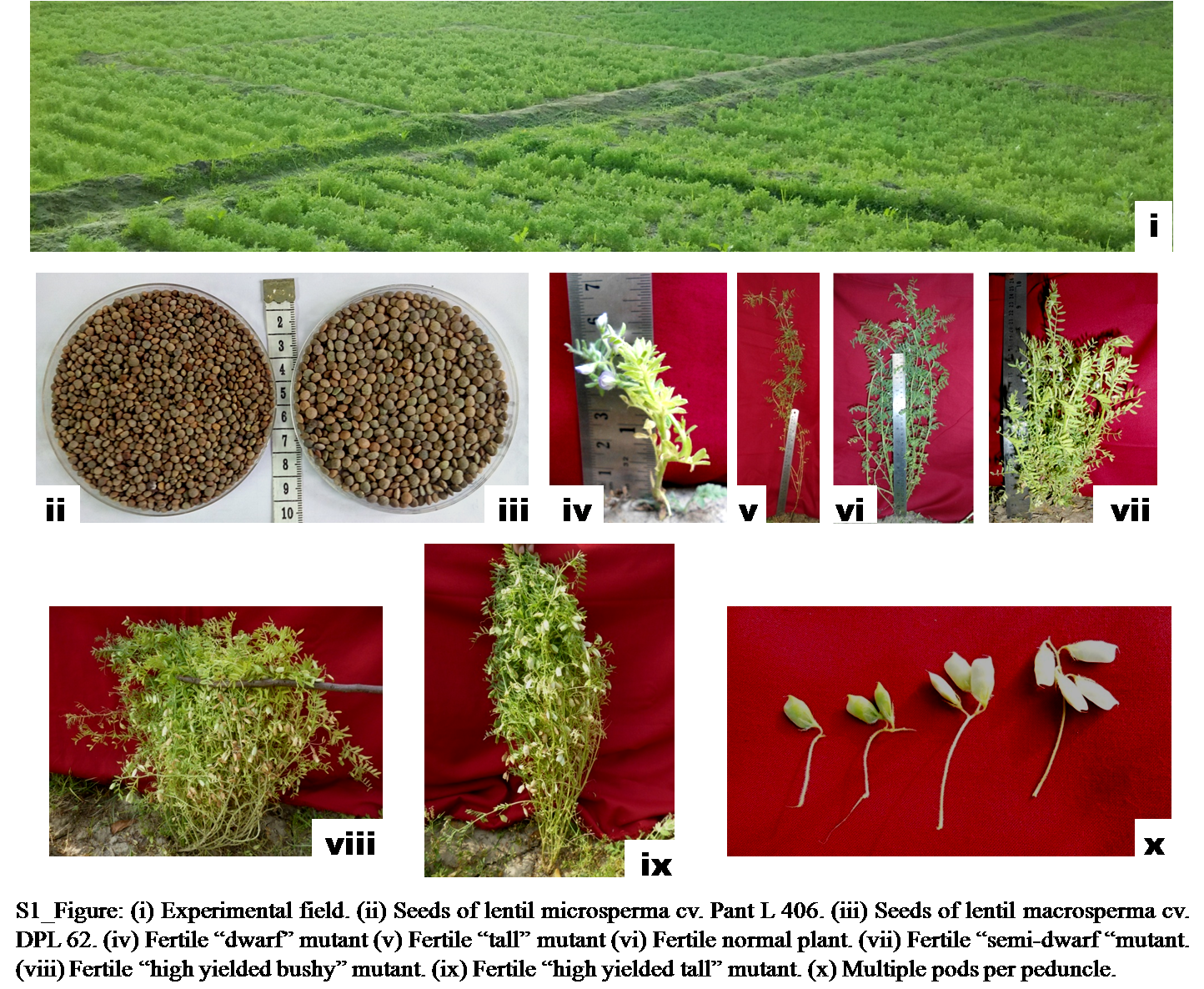

Supplement: S2 Fig — (i) Experimental field. (ii) Seeds of lentil microsperma cv. Pant L 406. (iii) Seeds of lentil macrosperma cv. DPL 62. (iv) Fertile “dwarf” mutant (v) Fertile “tall” mutant (vi) Fertile normal plant. (vii) Fertile “semi-dwarf “mutant. (viii) Fertile “high yielded bushy” mutant. (ix) Fertile “high yielded tall” mutant. (x) Multiple pods per peduncle. (TIF) [file pone.0184598.s006.tif]
